# Management of Rectal Wall Abscess: A Rare Case Report

**DOI:** 10.7759/cureus.50759

**Published:** 2023-12-19

**Authors:** Muhammad Talal Nasir, Kehkashan Anwar, Hareem Hussain, Mamoon Solkar, Sana Ullah

**Affiliations:** 1 General Surgery, Tameside and Glossop Integrated Care NHS Foundation Trust, Manchester, GBR; 2 Surgery, Tameside and Glossop Integrated Care NHS Foundation Trust, Manchester, GBR; 3 General and Colorectal Surgery, Tameside and Glossop Integrated Care NHS Foundation Trust, Manchester, GBR

**Keywords:** post fistulectomy, perianal disease, perianal pain, endoscopic drainage, rectal wall abscess

## Abstract

A 34-year-old male presented with spontaneous rectal pain. He was diagnosed with a posterior rectal wall abscess 7 cm proximal to the anal verge that was confirmed on an MRI (magnetic resonance imaging) pelvis. This abscess was drained through a transrectal route. Rectal wall abscess is an exceedingly rare pathology compared to perianal abscess; therefore, this case highlights the key aspects of its management.

## Introduction

Perianal abscesses and perianal fistula are common hospital presentations. Most (90%) of these abscesses develop as a result of infection of the anal crypto-globular glands, which are usually located posteriorly in the intersphincteric plane [1,2]. Rectal wall abscess is uncommon and requires a high index of clinical suspicion to diagnose it. Clinicians should be vigilant to this pathology as it may be dismissed as a simple perianal abscess. We describe the features and management of such pathology in the following case report.

## Case presentation

**Figure 1:**
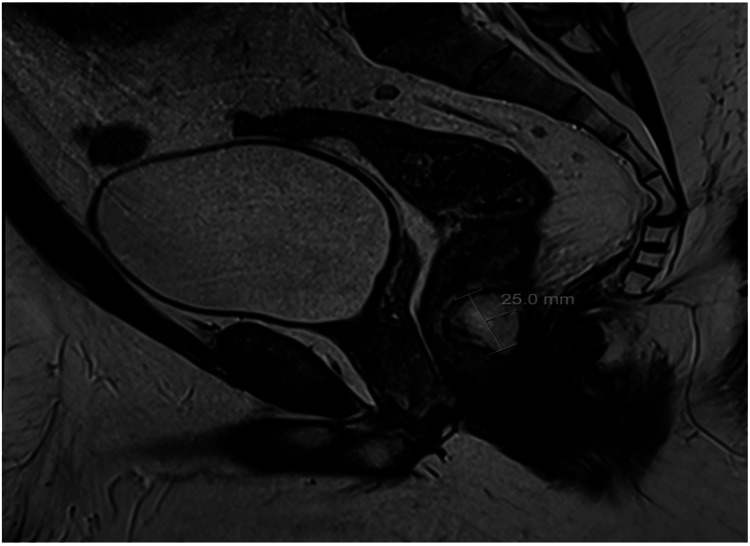
Rectal wall abscess 25 mm in diameter on T2-weighted images on MRI (sagittal view).

**Figure 2:**
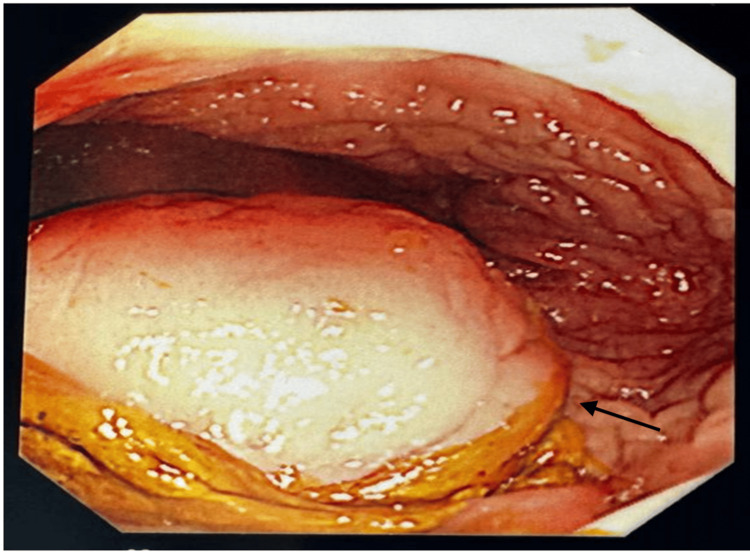
Flexible sigmoidoscopy view of the rectum and abscess with smooth and intact mucosa.

**Figure 3:**
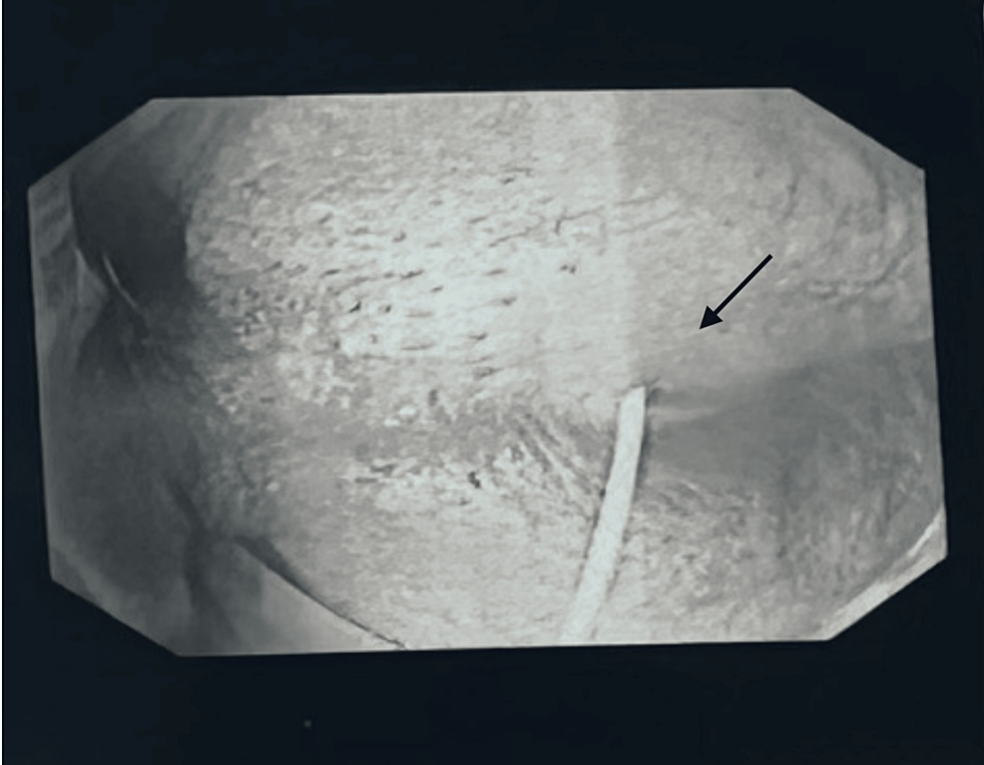
A small sinus at 1-2 o'clock position with a plastic cannula. No communication of this sinus with rectal abscess was identified.

A 34-year-old male presented with spontaneous rectal pain. The patient had a background of perianal fistula at the six o’clock position in 2021 for which he underwent examination under anaesthesia and open fistulectomy. Two years later, he re-presented with perianal pain, tenesmus, and raised inflammatory markers (WCC 14.3, CRP 93). On examination, per rectal examination, revealed a hard mass with a smooth surface at the 6-7 o’clock position. Based on that, an MRI of the pelvis was performed that showed a 25 mm abscess within the posterior wall of the lower rectum. It was 7 cm from the anal verge and far superior to the anal canal (Figure 1). The management options were discussed with the patient, and, subsequently, an examination under anaesthesia (EUA) for diagnostic and therapeutic purposes was arranged. EUA of the anorectum and a flexible sigmoidoscopy revealed the abscess as described. There was no visible sinus, and the mucosa was smooth, intact, and non-inflamed (Figure 2). Aspiration of the abscess under flexible sigmoidoscopy was attempted but was unsuccessful because of the presence of a thick abscess wall. It was ultimately drained using an Eisenhammer retractor and proctoscope. The wall of the abscess with rectal mucosa and pus swab was sent out for histopathology and microbiological analysis. The perianal area also revealed another small sinus at a 2 o'clock position with no evidence of a drainable abscess. Diluted methylene blue dye was injected with a pink cannula catheter into this sinus, but no communication was identified in the anorectum (Figure 3). No fistula opening was identified at the 6 o’clock position. No additional rectal or distal sigmoid pathology was identified on flexible sigmoidoscopy. The patient made an uneventful postoperative recovery and was discharged home. Subsequent culture reports of pus were negative for bacteria and tuberculosis. The histology of the specimen revealed normal colonic mucosa, a fragment showing marked ischemia and ulcerated features in keeping with the wall of an abscess. There was no evidence of granulomata, dysplasia, or malignancy.

**Figure 4:**
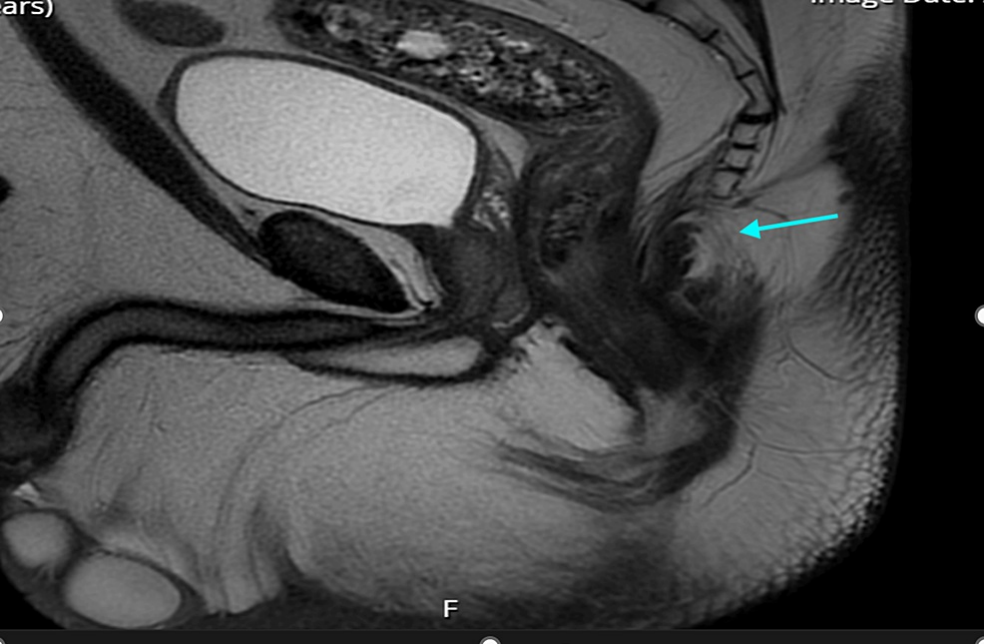
Postoperative (six weeks) MRI. No evidence of residual abscess or fistulae in the perianal and rectal areas.

After six weeks, a repeat MRI scan was performed that did not show any further pathology, residual abscess, or presence of perianal fistula (Figure 4). Additionally, all the previous and latest scans were reviewed again by a gastrointestinal radiologist in an X-ray meeting. There was no evidence of a fistula before the operation or afterwards. Afterwards, the patient was reviewed in the follow-up clinic where they were found to be asymptomatic since surgery. The patient was reassured and discharged.

## Discussion

Abscess formation in the perianal region is common, and there is a strong association of perianal abscess with perianal fistulae [1,2]. Such abscesses may also extend deep into the ischiorectal space. A PubMed and Google Scholar search was performed using the keywords “Rectum and Abscess,” but no results were found in the literature regarding "rectal wall abscess." Perianal abscesses and rectal wall abscesses may share the same etiology: an obstructed anal gland causing infection resulting in collection. In this case, we postulate that there may have been a blockage caused by the previous fistulectomy, resulting in scarring and subsequent abscess formation. The symptoms of rectal wall abscess were similar to those of perianal abscess, which includes perianal pain, painful defecation, and tenesmus. The exclusion of anal and low rectal cancers and polyps is an important aspect of their management. Other differentials include mucocele, inflammatory bowel disease, neurofibroma, GIST, and leiomyoma [3]. Clinical findings and background of previous fistula treatment in our case led to the decision to perform an MRI scan rather than a flexible sigmoidoscopy initially. Our patient had no history of smoking or diabetes, which are shown to have associations with perianal abscess or fistula [4,5].

Management of a submucosal rectal wall abscess is dependent on its size, position, distance from the anus, and etiology. Small or asymptomatic rectal abscesses may be treated with conservative measures or with antibiotics alone. Conversely, symptomatic and sizeable abscesses generally require surgical intervention. Low-lying abscesses may be accessible with general surgical instruments; however, more proximal abscesses may require endoscopic instruments or a transanal minimally invasive surgery (TAMIS) kit for their surgical drainage. Abscesses of the retroperitoneal area or out with the rectal wall may be suitable for drainage under CT guidance using a transgluteal approach [6]. We discussed this case with our radiology colleagues and because of the submucosal and spontaneous nature of the abscess, we decided to proceed with surgical drainage. Careful consideration of all aspects of patient care and available resources should be explored on a case-to-case basis. X-ray meetings/multidisciplinary team meetings are good forums to discuss these cases for decision-making and shared learning.

## Conclusions

Rectal wall abscess is a rare pathology that has not been reported before in the literature. We assume that this was secondary to the previously excised fistula tract owing to its anatomical position and previous surgical history. Clinicians should be aware of potential causes and management options for such cases.
